# Protein‐losing enteropathy as the first presentation of systemic lupus erythematosus: A case report from Sudan

**DOI:** 10.1002/ccr3.7314

**Published:** 2023-05-10

**Authors:** Elham Abdalla, Noon Mohymeed, Abdelsalam M. A. Nail, Rayan Ali Tonga, Mohammed Alfatih, Mohannad Abdalfdeel Almahie Shaban, Hassan Eltoum

**Affiliations:** ^1^ Department of Internal Medicine Bahri University Khartoum Sudan; ^2^ Department of Internal Medicine Omdurman Islamic University Khartoum Sudan; ^3^ Department of Internal Medicine Sudan Medical Specialization Board Khartoum Sudan; ^4^ Faculty of Medicine Alzaiem Alazhari University Khartoum Sudan

**Keywords:** ascites, protein‐losing enteropathy, systemic lupus erythematosus, tuberculosis

## Abstract

**Key Clinical Message:**

In low‐ and middle‐income countries, protein‐losing enteropathy is a diagnosis of exclusion. SLE should be on the list of differential diagnoses of protein‐losing enteropathy, especially if the patient had a long history of GI symptoms and ascites.

**Abstract:**

Protein‐losing enteropathy can rarely be the initial presentation of systemic lupus erythematosus (SLE). Protein‐losing enteropathy is a diagnosis of exclusion in low‐ and middle‐income countries. Protein‐losing enteropathy in SLE should be in the list of differential diagnosis of unexplained ascites, especially if patient had long history of gastrointestinal symptoms. We present a case of 33 years old male with long standing gastrointestinal symptoms and diarrhea attributed previously to irritable bowel syndrome. Presented with progressive abdominal distension, and diagnosed with ascites. Workup for him showed leucopenia, thrombocytopenia, hypoalbumenemia, elevated inflammatory markers (ESR 30, CRP 6.6), high cholesterol level (306 mg/dL), normal renal profile and normal urine analysis. Ascitic tab pale yellow with SAAG 0.9 and positive for adenosine deaminase (66 u/L) sugesstive for tuberculous peritonitis although quantitative PCR and geneXpert for MBT was negative. Antituberculous treatment was started and his condition deteriorated, immediately antituberculous was withdrawal. Further tests revealed positive serology for ANA (1:320 speckled pattern) with positive anti‐RNP/Sm, positive anti‐Sm antibodies. Complements level were normal. He started immunosuppressive therapy (prednisolone 10 mg/day, hydroxychloroquine 400 mg/day, azathioprine 100 mg/day). In addition, his condition is improved Diagnosis was made as SLE with Protein‐losing enteropathy based on hypoalbumenemia (with exclusion of renal loss of protein), ascites, hypercholesrtolemia and exclusions of other mimics as explained later. As well as positive response to immunosuppressive medications. Our patient diagnosed clinically as SLE with protein‐losing enteropathy. Protein‐losing enteropathy in SLE is challenging in diagnosis because of its rarity as well as limitations in its diagnostic tests.

## 1 INTRODUCTION

Systemic lupus erythematosus (SLE) is a prototypic multisystem autoimmune illness with an unexplained origin that disproportionately affects women of reproductive age. It has a strong genetic predisposition along with environmental factors, which act as triggers for loss of tolerance to self‐antigen, leading to antibody production and immune complex formation that can affect any part of the body and lead to different manifestations.^1^ Protein‐losing enteropathy is rare but has been reported as the first presentation of systemic lupus erythematous, characterized by loss of serum protein from the gastrointestinal tract.^2^ Excessive loss of serum protein (hypoalbuminemia) can lead to fluid balance disorders, edema, and heart failure. Moreover, patients with hypoalbuminemia are vulnerable to infection.^3^

## 2 PATIENT INFORMATION AND EXAMINATION

Our patient is a 33‐year‐old Sudanese male who presented 4 months ago complaining of dizziness and headache. No other symptoms at that time was diagnosed with malaria by blood film microscopic analysis and received antimalarial medications with no improvement. Then a few weeks later the patient develop depression and became in bad mood, seek for psychiatric consultation with little improvement.

Two months later the patient develops painless abdominal distension progressive over weeks, not associated with shortness of breath. He went to a tropical hospital and was diagnosed with mild ascites by the abdominal US for further workup and paracentesis was done and no definitive cause was elicited at that time and the decision was made to treat him with anti‐tuberculosis medications for suspected tuberculous peritonitis, due to positive ascitic fluid adenosine deaminase. Fifteen days later his condition deteriorated. He developed jaundice, fever, dyspnea, and increased abdominal swelling, immediately anti‐TB drugs were stopped and he received antibiotics according to blood culture. Then the patient was referred to us at the rheumatology unit because of further workup revealed that the serology profile was positive for ANA (1:320 speckled pattern) and positive anti‐RNP/Sm, positive anti‐Sm. complements level were normal at the tropical hospital.

Our medical interviews at the rheumatology unit revealed generalized weakness, fatigability, and weight loss. No joint pain and swelling, no morning stiffness, no skin rash, no photosensitive rash, no eyes symptoms nor sicca symptoms, and no Raynaud's symptoms. GIT revealed a 1‐year history of multiple attacks of abdominal pain and underwent upper GI endoscopy that showed gastritis.

Any cardiopulmonary and genitourinary symptoms were not observed. The nervous system revealed mood changes and depression and he is on antidepressive medications. He is not known to have any allergies and with history of smoking for 10 years and quit smoking with this current illness. He takes over‐the‐counter medications for IBS and gastritis. No autoimmune diseases run in the family. There is a history of hypertension in the family.

During admission, the patient's condition deteriorated and the mild ascites become massive with positive fluid thrill, and lower limb edema. Chest and cardiovascular examinations were normal. No oral ulcers. No joint deformities or evidence of synovitis or skin rash.

## 3 INVESTIGATIONS

Initial laboratory investigations were as follows: total white blood cell count, 2.9 cells/μL; hemoglobin, 15 g/dL; platelet count, 149 cells/μL; total bilirubin, 0.2 mg/dL; aspartate aminotransferase, 22 IU/L; alanine aminotransferase, 12 IU/L; serum total protein, 4.9 g/dL; serum albumin, 1.5 g/dL; serum globulin, 3.4 g/dL; then after starting antituberculous his tests show raising in serum bilirubin, 3.3 mg/dL; direct bilirubin, 2.5 mg/dL; indirect bilirubin, 0.8 mg/dL; ALK, 56μ/L; AST, 132μ/L; ALT, 29μ/L; blood urea nitrogen, 36 mg/dL serum creatinine, 1.0 mg/dL; Na, 128 mmoL/L; K, 3.7 mmoL/L; serum phosphate, 2.3 mg/dL; Ca, 6.9 mg/dL and after correction Ca was 8.9 mg/dL due to hypoalbuminemia; total cholesterol, 306 mg/dL; C‐reactive protein (CRP), 6.6 mg/dL; erythrocyte sedimentation rate, 30 mm/h. The viral screening was negative and urine analyses were normal. He received antibiotics (Meropenem 1 g IV every 8 h for 7 days) for *Klebsiella* infection according to culture and sensitivity at a tropical hospital.

Autoantibody profiles showed positive ANA (1:320 speckled pattern) anti‐RNP/Sm, and positive anti‐Sm, and the diagnosis of SLE was established based on the ACR and EULAR criteria for the classification of SLE (score 15 points).

Chest x‐ray showed no abnormalities, echocardiography showed LVEF 57% and mild pericardial effusion. The US of the abdomen done early in admission showed normal liver architecture, mild ascites, diffuse gallbladder wall thickness, and mildly enlarged spleen but no evidence of portal hypertension. Later in admission, the patient condition deteriorated and ascites become massive with fluid thrill clinically detected, and lower limb edema.

Paracentesis was performed and the fluid analysis showed pale yellow ascitic fluid; albumin, 2.4 g/dL; sugar content, 99 mg/dL; LDH, 249μ/L; with no nucleated cells or acellular deposition was seen. Ascitic AFB was negative, quantitative PCR for mycobacterium tuberculosis (MTB) was negative, and ascitic fluid gene Xpert for MTB was negative as well. ADA ascitic fluid was significant (66μ/L) and suggestive of tuberculosis, based on ADA ascitic fluid the patient started antituberculous with further deterioration then antituberculous was withdrawn and Ascites were considered to be caused by hypoproteinemia. The serum ascitic albumin gradient (SAAG) was 0.9. Lupus nephritis was unlikely in the absence of any significant abnormalities in kidney functions and clear urinalysis. Other causes of edema and ascites such as liver, heart failure, or thyroid dysfunction were also unlikely, the patient workup for evaluating the possibility of PLE was necessary. The patient was requested to do CT abdomen and endoscopy with biopsy to exclude other causes, he was offered counseling many times on how these investigations would help with the management unfortunately because of his financial difficulties and as we are in a resource‐limited country, moreover advanced tests were not available in the center, PLE was identified clinically as the cause of hypoproteinemia found in this patient.

After the diagnosis of PLE as a presentation of SLE, corticosteroids were prescribed and the patient clinical improvement and was discharged with an outpatient prescription of prednisone 10 mg/day, azathioprine 50 mg twice/day and hydroxychloroquine 200 mg twice/day. The patient was followed up in the referred clinic and he responded to the treatment over the next few months as evidenced by the rise in serum albumin subsequently. He is well up to date and returns to his work with no significant complications. No further workup was done (like a CT abdomen and colonoscopy) as the patient's symptoms and conditions improved rapidly.

## 4 DISCUSSION

The present case describes a 33‐year‐old male, diagnosed clinically with Protein‐losing enteropathy as the first presentation of SLE.

In low‐ and middle‐income countries, PLE is a diagnosis of exclusion and should be considered in the list of differential diagnoses of any unexplained ascites. The diagnosis of protein‐losing enteropathy required 24 h fecal a1 antitrypsin level, MR lymphangiography, and technetium 99 albumin scintigraphy.^3^ Unfortunately, these investigations were not available at our hospital to make a final diagnosis. However, we could confidently conclude that PLE was responsible for hypoalbuminemia as the good response of serum albumin following steroid treatment.

Pseudo‐pseudo Meigs syndrome (PPMS) should be in the differential diagnosis of ascites in SLE but in PPMS, there will be no gastrointestinal symptoms such as diarrhea and the ascites will be exudates in nature accompanied also by pleural effusion. Here in our case, we exclude PPMS by the prolonged history of diarrhea and gastrointestinal symptoms which support more protein‐losing enteropathy. Lupus peritonitis should be in the differential diagnosis of ascites in SLE but the abdomen examination will show tenderness and the serum protein will be normal, unlike our case with no tenderness and very low serum albumin which suggest PLE as the culprit cause in such a case. Also, hypercholesterolemia in our case supports our diagnosis of PLE.

SLE is the most common autoimmune disease, and it has a wide range of clinical presentations with a remitting and relapsing course. SLE commonly affects the gastrointestinal tract and most cases can be life‐threatening. The most common cause is lupus mesenteric vasculitis, followed by protein‐losing enteropathy, intestinal pseudo‐obstruction, acute pancreatitis, and others such as celiac disease, inflammatory bowel diseases, etc.^4^

Our patient had protein‐losing enteropathy as an initial presentation of SLE that presented as subacute ascites. Serositis in SLE is associated with high morbidity, and its prevalence ranges between 11% and 54%.^5^, ^6^

Ascites were formerly considered to exclusively manifest in cases of nephrotic syndrome, Budd‐Chiari syndrome, and protein‐losing enteropathy.^6^, ^7^ Nonetheless, 10% of SLE patients also experience ascites.^8^ Ascites that accompany acute peritonitis as the initial presentation for lupus are incredibly uncommon, and to our knowledge, a few cases in the literature have mentioned this presentation in juvenile patients, while other related cases have also occurred in well‐known SLE patients.^7^, ^9^ In the literature, active lupus disease, fever, and high d‐dimer are significantly associated with serositis.^10^ Immune complex deposition in the peritoneum with activation of cytokines, IL‐1β, IL6, TNF‐α, INF‐ɣ. Vasculitis of peritoneal vessels may contribute to the pathogenesis of lupus peritonitis.^11^

Although an association between SLE and protein‐losing enteropathy has been recognized, there have been less than 200 cases documented in world literature^2^, ^12^ there are no documented cases of such an association in Sudan literature.

SLE presenting as protein‐losing enteropathy has been reported even less commonly. Vasculitis of mesenteric vessels, inflammatory reactions along the digestive tract: mucosal edema, alterations in capillary permeability, and lymphangiectasia have been postulated as the pathogenic mechanisms.^4^

Zhen Chen 2014 reported that PLE in SLE had a significant association with greater frequencies of anti‐Sm, anti‐SSA, and hypocomplementemia, and higher cholesterol levels compared with the control group.^3^, ^12^

## AUTHOR CONTRIBUTIONS

**Elham Abdalla:** Conceptualization; data curation; investigation; methodology; project administration; writing – original draft; writing – review and editing. **Noon Mohymeed:** Conceptualization; data curation; methodology; writing – original draft; writing – review and editing. **Abdelsalam M.A. Nail:** Writing – original draft; writing – review and editing. **Rayan Ali Tonga:** Writing – original draft; writing – review and editing. **Mohammed Alfatih:** Conceptualization; project administration; writing – original draft; writing – review and editing. **Mohannad Abdalfdeel Almahie Shaban:** Writing – original draft; writing – review and editing. **Hassan Eltoum:** Conceptualization; project administration; supervision; writing – original draft; writing – review and editing.

## FUNDING INFORMATION

No funds.

## CONFLICT OF INTEREST STATEMENT

The authors have no conflict of interest to declare.

## CONSENT

Written informed consent was obtained from the patient to publish this report in accordance with the journal's patient consent policy.

## Data Availability

On reasonable request, the corresponding author will provide the data that support the findings of this study.
